# Transferability of Single‐ and Cross‐Tissue Transcriptome Imputation Models Across Ancestry Groups

**DOI:** 10.1002/gepi.22611

**Published:** 2025-01-15

**Authors:** Inti Pagnuco, Stephen Eyre, Magnus Rattray, Andrew P. Morris

**Affiliations:** ^1^ Centre for Genetics and Genomics Versus Arthritis, Centre for Musculoskeletal Research, Division of Musculoskeletal and Dermatological Sciences The University of Manchester Manchester UK; ^2^ Division of Informatics, Imaging and Data Sciences The University of Manchester Manchester UK

**Keywords:** ancestry, expression quantitative trait loci, imputation, tissue, transcriptome‐wide association study, transferability

## Abstract

Transcriptome‐wide association studies (TWAS) investigate the links between genetically regulated gene expression and complex traits. TWAS involves imputing gene expression using expression quantitative trait loci (eQTL) as predictors and testing the association between the imputed expression and the trait. The effectiveness of TWAS depends on the accuracy of these imputation models, which require genotype and gene expression data from the same samples. However, publicly accessible resources, such as the Genotype Tissue Expression (GTEx) Project, are biased toward individuals of European ancestry, potentially reducing prediction accuracy into other ancestry groups. This study explored eQTL transferability across ancestry groups by comparing two imputation models: PrediXcan (tissue‐specific) and UTMOST (cross‐tissue). Both models were trained on tissues from the GTEx Project using European ancestry individuals and then tested on data sets of European ancestry and African American individuals. Results showed that both models performed best when the training and testing data sets were from the same ancestry group, with the cross‐tissue approach generally outperforming the tissue‐specific approach. This study underscores that eQTL detection is influenced by ancestry and tissue context. Developing ancestry‐specific reference panels across tissues can improve prediction accuracy, enhancing TWAS analysis and our understanding of the biological processes contributing to complex traits.

## 1 Introduction

Genome‐wide association studies (GWAS) have successfully identified regions of the genome associated with complex human traits and diseases. However, the identification of causal genes and the understanding of the biological mechanisms underpinning these associations often remain unclear (Visscher et al. 2012). An important factor contributing to this challenge is the considerable proportion of statistically significant GWAS signals that map to noncoding regions of the human genome (Maurano et al. 2012). Previous studies have indicated that many of these GWAS signals may act as regulators of gene transcript abundance, referred to as expression quantitative trait loci (eQTL) (Nicolae et al. 2010). Therefore, focusing on the identification of eQTL capable of modulating gene expression could provide additional insights into the impact of these GWAS signals on complex traits and diseases and could contribute to a deeper understanding of the mechanisms through which genotype influences phenotype (Albert and Kruglyak 2015).

The emergence of resources such as the Genotype Tissue Expression (GTEx) Project (Ardlie et al. 2015), which provides matched genotype and gene expression data across a range of tissues, has prompted the development of novel methodology to investigate relationships between genotype, gene expression, and traits, referred to as transcriptome‐wide association studies (TWAS). This innovative methodology first estimates the aggregate effect of eQTL on the regulation of target genes in a single tissue or across multiple tissues in a sample of individuals with both genotype and expression data available (such as GTEx) in a process referred to as training. These models are then used to impute gene expression in an independent sample of individuals in which genotype data are available and examine the association with a given trait. As part of TWAS software packages, multiple statistical approaches have been developed to impute gene expression using aggregate eQTL models as predictors (Gamazon et al. 2015; Gusev et al. 2016; Hu et al. 2019). Most TWAS approaches evaluate the aggregate effect on gene expression of cis‐regulatory variants (i.e., mapping within 1 Mb of the transcription start and stop sites of the gene) but are based on different hypotheses regarding the tissue context dependency of the eQTL regulatory mechanism. For example, PrediXcan (Gamazon et al. 2015) uses an elastic‐net regression model to detect tissue‐specific eQTL, while UTMOST (Hu et al. 2019) employs group‐lasso and simultaneously explores all tissues to identify cross‐tissue eQTL.

Achieving accurate transcriptome imputation requires a statistical model whose assumptions closely align with the true genetic architecture of gene expression. However, a significant limitation arises as the currently available resources for training eQTL models, such as GTEx, predominantly consist of individuals of European ancestry, which may impact on the transferability of TWAS into other ancestry groups. Indeed, numerous studies have presented compelling evidence suggesting that GWAS findings often do not generalize from European ancestry populations into other ancestry groups. This lack of transferability of GWAS signals is attributed to variation in allele frequencies and linkage disequilibrium (LD) patterns among ancestry groups (Adeyemo and Rotimi 2010; Carlson et al. 2013; Li and Keating 2014), which would be expected to also impact on the performance of ancestry‐specific eQTL models and TWAS (Kelly, Hansen, and Tishkoff 2017).

In this study, we compared the performance of European ancestry‐derived transcriptome imputation models for prediction of gene expression into European ancestry individuals and admixed African American individuals from the GTEx Project. To assess the impact of the tissue context dependency of our findings, we considered imputation models for a total of 49 tissues using both PrediXcan and UTMOST.

## 2 Methods

### 2.1 GTEx Project Data and Quality Control

Approved access for whole‐genome sequencing and gene expression data for 953 individuals from the GTEx Project was granted through dbGaP (accession number phs000424.v9.p2).

**Figure 1:**
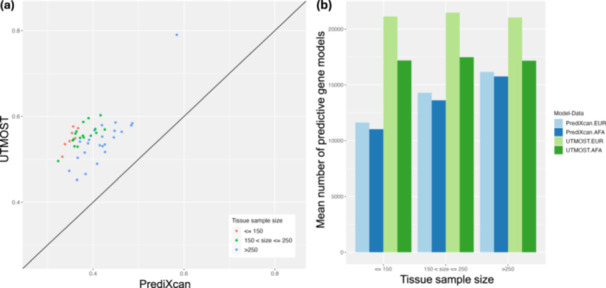
Numbers of gene expression imputation models trained in 587 European ancestry individuals and subsequently predictive in 111 European ancestry individuals and 111 African American individuals, using single tissue (PrediXcan) and cross‐tissue (UTMOST) approaches. Gene expression data were interrogated for 49 tissues for 38,539 autosomal genes. (a) Proportion of genes for which imputation models could be trained for each tissue. Kidney cortex tissue was excluded from the plot because of the small number of imputation models trained using PrediXcan. (b) Mean number of trained gene models that were predictive in European ancestry and African American test data sets.

**Figure 2:**
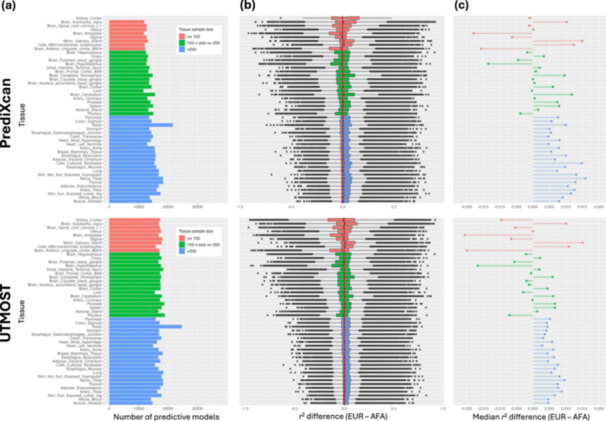
Tissue‐specific improvement in prediction accuracy of gene expression imputation models for European ancestry (EUR) test data set over African American (AFA) test data set using single tissue (PrediXcan) and cross‐tissue (UTMOST) approaches. Gene expression data were interrogated for 49 tissues for 38,539 autosomal genes. (a) Number of trained gene models that were predictive in both test data sets. (b) Distribution of difference in squared Pearson's correlation (*r* ^2^) between imputed and observed gene expression for EUR test data set over AFA test data set. (c) Median difference in squared Pearson correlation (*r* ^2^) between imputed and observed gene expression for EUR test data set over AFA test data set.

For genotype quality control, we first excluded individuals with a call rate < 99.9%. The distribution of autosomal heterozygosity was bimodal, reflecting the ancestral diversity of individuals (Supporting Information S1: Figure 1). We extracted two subsets of individuals according to autosomal heterozygosity. First, we extracted individuals with autosomal heterozygosity of 0.045−0.050, which when compared to self‐reported identifiers reflected European ancestry. Second, we extracted individuals with autosomal heterozygosity of 0.060−0.080, which when compared to self‐reported identifiers reflected African Americans. Within each subset of individuals, SNVs with call rate < 90% or Hardy−Weinberg equilibrium exact test *p* < 10^−6^ were excluded. Within each subset, we then conducted principal components analysis using PLINK v1.9 after excluding SNVs with minor allele frequency (MAF) < 5%, removal of high LD regions, and LD pruning (*r* ^2^ < 0.05). After excluding outliers on the first two principal components, we retained 689 individuals of European ancestry and 111 African American individuals (Supporting Information S1: Figure 2).

Within each subset of individuals, the normalized tissue‐specific gene expression data underwent adjustments to eliminate potential confounding effects from sex, sequencing platform (HiSeq. 2000 or HiSeqX), library construction protocol (PCR‐based or PCR‐free), the first two principal components, and the top probabilistic estimation of expression residuals (PEER) factors (Stegle et al. 2010). Following the recommendations from previous studies (Ardlie et al. 2015), the number of PEER factors included depending on the number of samples per tissue (15 PEER factors for tissues with less than 150 samples, 30 PEER factors for tissues with between 150 and 249 samples, 45 PEER factors for tissues with between 250 and 349 samples, and 60 PEER factors for tissues with at least 350 samples).

### 2.2 Transcriptomic Imputation Gene Model Training

We randomly selected 587 individuals of European ancestry for transcriptomic imputation model training. We trained single‐tissue PrediXcan gene models and cross‐tissue UTMOST gene models using genotype and normalized gene expression data for 49 tissues for 38,539 autosomal genes. The sample sizes for the different tissues ranged from 73 (for kidney cortex) to 706 (for skeletal muscle) (Supporting Information S1: Table 1). We considered only those SNVs mapping within 1 Mb of the transcription start and stop sites of the gene and excluded SNVs with ambiguous alleles (AT or GC) or with MAF < 1%. We used both methods with default settings.

### 2.3 Testing Trained Gene Models in European Ancestry Individuals and African American Individuals

For each gene, we used the single‐tissue PrediXcan trained models and cross‐tissue UTMOST trained model to impute gene expression in each of the 49 tissues using genotype data for: 111 individuals of European ancestry (not selected for training); and 111 African American individuals. For each gene/tissue combination, we calculated the squared Pearson's correlation (*r* ^2^) between imputed and observed expression.

## 3 Results

### 3.1 Number of Trained and Predictive Gene Models Across Tissues

We began by comparing the number of gene models that were trained for PrediXcan and UTMOST for each tissue (Figure 1). A gene model is considered to be untrained if PrediXcan or UTMOST removes all SNV features for that gene during training (i.e., the sparse regression removed the effect of all features on the training data). Across tissues, UTMOST consistently demonstrated a higher number of trained gene models (mean 55.0%) when compared to PrediXcan (mean 38.6%). Moreover, the cross‐tissue approach implemented in UTMOST demonstrated less sensitivity to sample size variability across tissues than the single‐tissue approach implemented in PrediXcan. For example, in the tissue with the smallest sample size (kidney cortex, comprising 73 samples), PrediXcan resulted in < 0.1% trained gene models, while UTMOST achieved 55.4%. This would be expected because UTMOST considers all tissues simultaneously and can, therefore, take advantage of correlation in gene expression profiles across tissues to train gene models.

After training the gene models, the number of predictive gene models per tissue was compared between PrediXcan and UTMOST, and between European ancestry and African American test data sets (Figure 1, Supporting Information S1: Table 1). A trained gene model was not predictive if all the selected SNVs were monomorphic in the test data set. We note that a selected SNV in the European ancestry training data set can be monomorphic in the European ancestry test data set because of the smaller sample size. UTMOST consistently yielded a higher number of predictive gene models than PrediXcan across tissues in both test data sets. Notably, for PrediXcan, there was a trend for a larger number of predictive gene models for tissues with larger sample sizes. This bias was overcome in UTMOST because each gene model is trained across all tissues simultaneously. For both approaches, more gene models were predictive in the European ancestry test data set than in the African American test data set, with the difference between the test data sets more pronounced for UTMOST than for PrediXcan (Supporting Information S1: Table 1). This difference between test data sets would be expected because SNVs identified in a European ancestry training gene model are less likely to be polymorphic in an African American test data set than in a European ancestry test data set.

### 3.2 Transferability of Transcriptomic Imputation Across Ancestry Groups

We next considered the imputation accuracy of the trained gene models in the European ancestry and African American test data sets for each tissue by comparing predicted and observed gene expression using the squared Pearson's correlation (*r* ^2^). For comparisons, we considered only those gene/tissue combinations for which the gene model was predictive in both test data sets (Supporting Information S1: Figure 3).

For both PrediXcan and UTMOST, the tissue sample size impacts on the difference in prediction accuracy between test data sets (Figure 2, Supporting Information S1: Table 2). The median improvement in *r* ^2^ from African American to European ancestry test data sets was largest for tissues with the largest sample sizes. Furthermore, the variation (measured by the interquartile range) in improvement in *r* ^2^ from African American to European ancestry data sets was smallest for tissues with the largest sample sizes. Interestingly, for 10 out of 49 tissues in PrediXcan, and 15 out of 49 tissues in UTMOST, the median improvement in *r* ^2^ from African American to European ancestry test data sets was negative, indicating better performance of the trained gene model, on average, in the African American test data set, despite the mismatch in ancestry (Figure 2, Supporting Information S1: Table 2). However, these tissues typically had small sample sizes with considerable variation in the improvement in *r* ^2^. Across all tissues, the mean improvement in *r* ^2^ from African American to European ancestry test data sets was 0.00473 for PrediXcan and 0.00212 for UTMOST.

For each tissue, we also calculated the proportion of genes for which the imputation accuracy was greater in the European ancestry test data set than the African American test data set for both imputation approaches (Supporting Information S1: Table 3). For most tissues, particularly those with the largest sample sizes, *r* ^2^ was more often greatest in the European ancestry test data set for both PrediXcan and UTMOST. Across all tissues, the mean proportion of genes for which the imputation accuracy was greatest in the European ancestry test data set was 56.2% for PrediXcan and 53.9% for UTMOST.

### 3.3 Impact of Tissue Context Dependency on Transcriptomic Imputation Performance

We finally assessed the impact of tissue context dependency of eQTLs on gene expression imputation models by separately evaluating the performance of PrediXcan and UTMOST in the European ancestry and African American test data sets ancestry data sets. When both PrediXcan and UTMOST were predictive in each test data set, we compared the prediction accuracy of the trained gene models from the two approaches. Across tissues, the mean number of genes for which the trained gene model could be fitted with both approaches was 13,430 (34.8%) in the European ancestry test data set and was 10,552 (27.4%) in the African American test data set.

For both test data sets, the tissue sample size impacts on the difference in prediction accuracy between UTMOST and PrediXcan (Supporting Information S1: Figure 4, Supporting Information S1: Table 4). The median improvement in *r* ^2^ from PrediXcan to UTMOST was largest for tissues with the smallest sample sizes. However, the variation (measured by the interquartile range) in improvement in *r* ^2^ from PrediXcan to UTMOST was also largest for tissues with the smallest sample sizes. For 4 out of 49 tissues in the European ancestry test data set, and 3 out of 49 tissues in the African American test data set, the median improvement in *r* ^2^ from PrediXcan to UTMOST was negative, indicating poorer performance of cross‐tissue imputation than single‐tissue imputation (Supporting Information S1: Figure 4, Supporting Information S1: Table 4). These tissues mostly had large sample sizes, where there was less to gain from the cross‐tissue modeling than for tissues with small sample sizes. Across all tissues, the mean improvement in *r* ^2^ from PrediXcan to UTMOST was 0.00473 in the European ancestry test data set and 0.00207 in the African American test data set.

For each tissue, we also calculated the proportion of genes for which the imputation accuracy was greater for UTMOST than PrediXcan in the two test data sets (Supporting Information S1: Table 5). For most tissues, particularly those with the smallest sample sizes, *r* ^2^ was more often greatest for UTMOST in both test data sets. Across all tissues, the mean proportion of genes for which the imputation accuracy was greatest for UTMOST was 55.1% in the European ancestry test data set and 52.6% in the African American test data set.

## 4 Discussion

Many methods have been developed for imputing gene expression by utilizing eQTLs as predictors (Mai et al. 2023). Training these statistical models requires matched genotype and gene expression data from the same set of individuals. However, most publicly accessible resources, such as the GTEx Project, predominantly consist of samples from individuals of European ancestry. Relying on these resources may result in inaccurate gene expression prediction models for individuals from other ancestry groups. This study explored the transferability of eQTL across different ancestry groups by comparing the performance of two gene expression imputation model approaches: a single‐tissue model (implemented by PrediXcan) and a cross‐tissue model (implemented by UTMOST). Both models were trained using a data set exclusively composed of samples from European ancestry individuals and were subsequently tested in data sets comprising European ancestry individuals and admixed African Americans. Whilst many single‐tissue and cross‐tissue methods have been developed (Khunsriraksakul et al. 2022; Mai et al. 2023), our objective was to evaluate transferability of gene models across ancestry groups with “baseline” approaches, rather than provide a detailed comparison of methods.

We first explored how genetic ancestry influences model performance by comparing the accuracy of gene expression imputation models in the two test data sets. Across most tissues, both approaches demonstrated improved prediction accuracy in the European ancestry test data set. The discrepancy in performance was greater for the single‐tissue approach implemented in PrediXcan than for the cross‐tissue approach implemented in UTMOST. It is important to emphasize that our assessment of the transferability of trained gene models across ancestry groups is based only on the subset of models that are predictive in both test data sets. Consequently, the differences in prediction accuracy between ancestry groups are likely to be more pronounced than we report because the proportion of trained gene models that were predictive was lower in the African American test data set. Possible explanations for the difference in performance between test data sets include variations in LD patterns or allele frequencies across ancestry groups (Kelly, Hansen, and Tishkoff 2017; Mogil et al. 2018). Considering that the gene models were trained using only samples from individuals of European ancestry, our findings align with recent studies indicating that prediction accuracy tends to be higher when the training and test data sets share similar genetic ancestry (Gottlieb et al. 2017; Li et al. 2018; Mogil et al. 2018; Mikhaylova and Thornton 2019). Previous studies examining the performance of gene expression imputation models employed different cohorts for training and testing the model (Fryett et al. 2018; Li et al. 2018; Mikhaylova and Thornton 2019; Li et al. 2019), and differences in data processing across cohorts could influence comparisons (Fryett, Morris, and Cordell 2020). In this study, we mitigate disparities in data processing samples by exclusively using individuals from the GTEx Project for both training and testing the gene models. Furthermore, previous studies have focussed transferability assessments on single‐tissue imputation approaches (Mogil et al. 2018; Geoffroy, Gregga, and Wheeler 2020), whilst our results highlight that the lower prediction accuracy observed when training and test data sets are not matched for ancestry extends also to cross‐tissue contexts. Understanding the impact of ancestry is crucial to fully leverage the potential of cross‐tissue gene expression imputation models across diverse genetic ancestry groups.

We then assessed the impact of the tissue context dependency of eQTL by comparing the performance of PrediXcan and UTMOST within each of the European ancestry and African American test data sets. PrediXcan considers the effects of eQTL in the context of a single tissue, neglecting the potential multi‐tissue effects of eQTL. In contrast, UTMOST estimates the effects of eQTLs in a cross‐tissue context, allowing for the correlation in expression between tissues, and thereby increasing the likelihood of detecting multi‐tissue eQTL. In both European ancestry and African American test data sets, UTMOST demonstrated superior performance over PrediXcan across most tissues. Similar findings have been reported previously, but not in the context of comparisons across ancestry groups (Hu et al. 2019; Barbeira et al. 2020). The difference in performance between approaches diminished as the sample size of the tissues increased. Specifically, UTMOST exhibited lower sensitivity to smaller sample sizes than PrediXcan, and trained gene models that were predictive for a larger number of genes. UTMOST leverages information from all available tissues for each gene model, building on compelling evidence that regulatory mechanisms driving gene expression are shared across multiple tissues (Ardlie et al. 2015). It is worth noting that both modeling approaches are trained using local SNVs, in cis, without considering distal regulation. Previous cross‐tissue gene expression studies have confirmed that local regulation of gene expression is broadly shared across tissues, while distal regulation is notably tissue‐specific (Liu et al. 2017).

The GTEx Project currently provides matched genotype and expression data from 49 tissues, making it a valuable resource for training prediction models. In total, we considered 38,539 autosomal genes, for which gene models for fewer than 50% could be trained with either PrediXcan or UTMOST. Fewer trained gene models were predictive in the African American test data set than in the European ancestry test data set. This can be attributed to the eQTL detected in the model training on samples from individuals of European ancestry being monomorphic in the African American test data set. This mismatch of individuals between training and test data sets, referred to as “data set shift,” can impair the application of the fitted model in the test data set (Dockès, Varoquaux, and Poline 2021). One potential approach to improve the transferability of trained gene models would be to restrict SNV selection to those that have MAF > 0.5% in all ancestry groups in reference data sets such as the 1000 Genomes Project (1000 Genomes Project Consortium 2015) or the Human Genome Diversity Project (Li et al. 2008). Such an approach would increase the likelihood that SNVs in trained gene models would be polymorphic in test data sets, irrespective of ancestry. However, this approach would also be expected to decrease prediction accuracy in test data sets matched for ancestry with the training data set because ancestry‐specific SNVs contributing to the regulation of gene expression would be overlooked.

The poor transferability between ancestry groups of prediction models for gene expression have clear parallels with polygenic risk scores (PRS). The prediction accuracy of PRS decreases as the genetic distance increases between training data set and test data set (Kachuri et al. 2024). To overcome this challenge, recent developments have introduced multi‐ancestry PRS methods (Zhang et al. 2023; Zhang et al. 2024), which enhance transferability and robustness by incorporating genetic data from more diverse populations, resulting in more accurate and representative predictions. In the same way, novel TWAS methods have been proposed to integrate eQTL reference data from multi‐ancestry populations. For example, TESLA (Chen et al. 2023) and MATS (Knutson and Pan 2023) adapt pre‐trained gene models by incorporating ancestry‐specific adjustments after the initial training phase. In contrast, METRO (Li et al. 2022) integrates gene models constructed across different ancestry groups by jointly estimating the ancestry‐specific effects of SNVs on both gene expression and disease/trait outcomes. These approaches enhance TWAS power and generalizability by leveraging multiple ancestry groups, but their use is limited by the current lack of genetic diversity in resources with matched genotype and gene expression data. Continued development in this area will be crucial for further improving the inclusivity and precision of genetic risk assessments across diverse populations.

In conclusion, our study has demonstrated that the performance of gene expression imputation methods can be influenced by both genetic ancestry and tissue context. Expanding resources with matched genotype and gene expression data to incorporate more individuals, especially from non‐European ancestry populations, and obtaining samples for tissues and cell types not currently included in the GTEx Project would enhance prediction accuracy (Arruda, Morris, and Zeggini 2024). Improved prediction accuracy across diverse ancestry groups would ultimately increase the power of TWAS and enhance the opportunities for the clinical translation of GWAS of common complex diseases for global populations.

## Conflicts of Interest

The authors declare no conflicts of interest.

## Supporting information

Supporting information.

Supporting information.

## Data Availability

The authors have nothing to report.
