# Supplementary material for: Transferability of Single‐ and Cross‐Tissue Transcriptome Imputation Models Across Ancestry Groups
Source: Genet Epidemiol. 2025 Jan 15;49(1):e22611. doi: 10.1002/gepi.22611 (PMC11734644; doi:10.1002/gepi.22611)
Supplement: Supplementary file 1 — Supporting information. [file GEPI-49-0-s002.pdf]

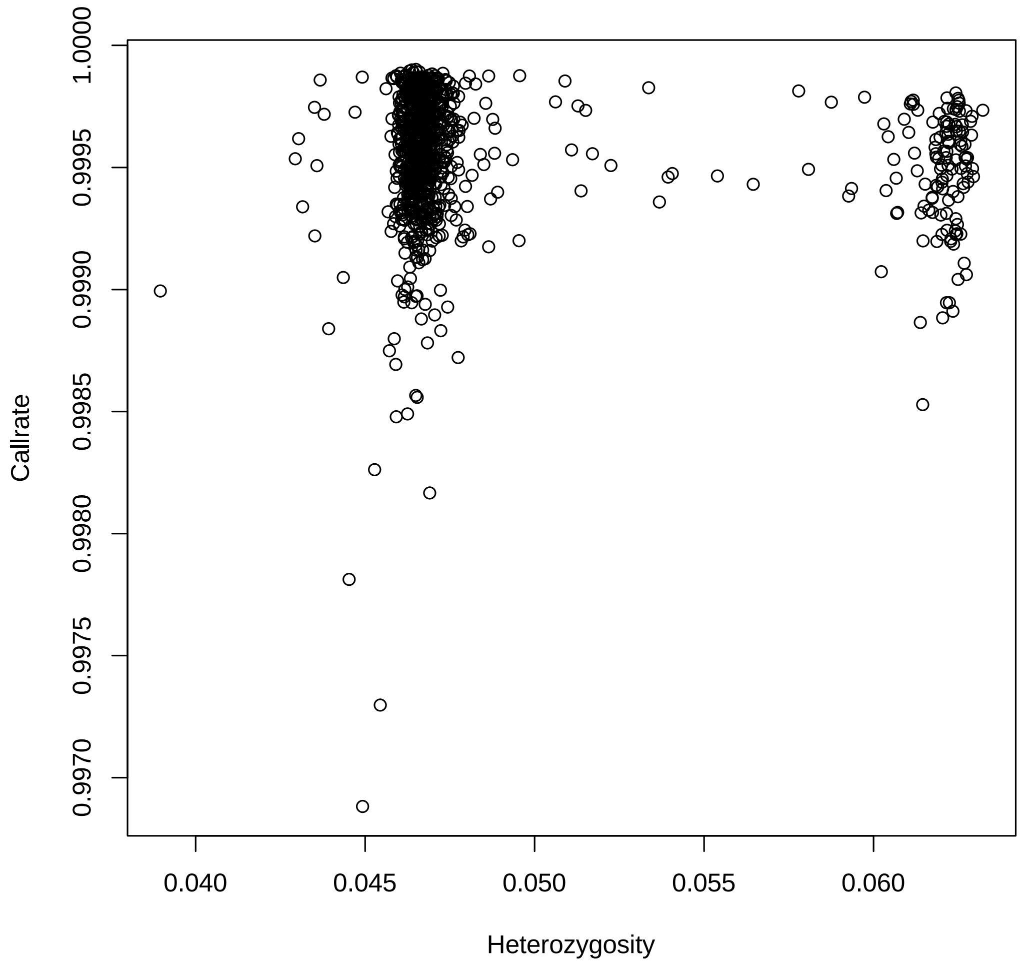

Supplementary Figure 1. Sample quality control of genotype data for 953 individuals from the GTEx Project. Each sample is plotted according to their call rate (on the y-axis) and autosomal heterozygosity (on the x-axis). We excluded individuals with call rate <99.9%. We first extracted individuals with heterozygosity of 0.045-0.050, which when compared to self-reported identifiers reflected European ancestry. We then extracted individuals with heterozygosity of 0.060-0.080, which when compared to self-reported identifiers reflected African Americans.

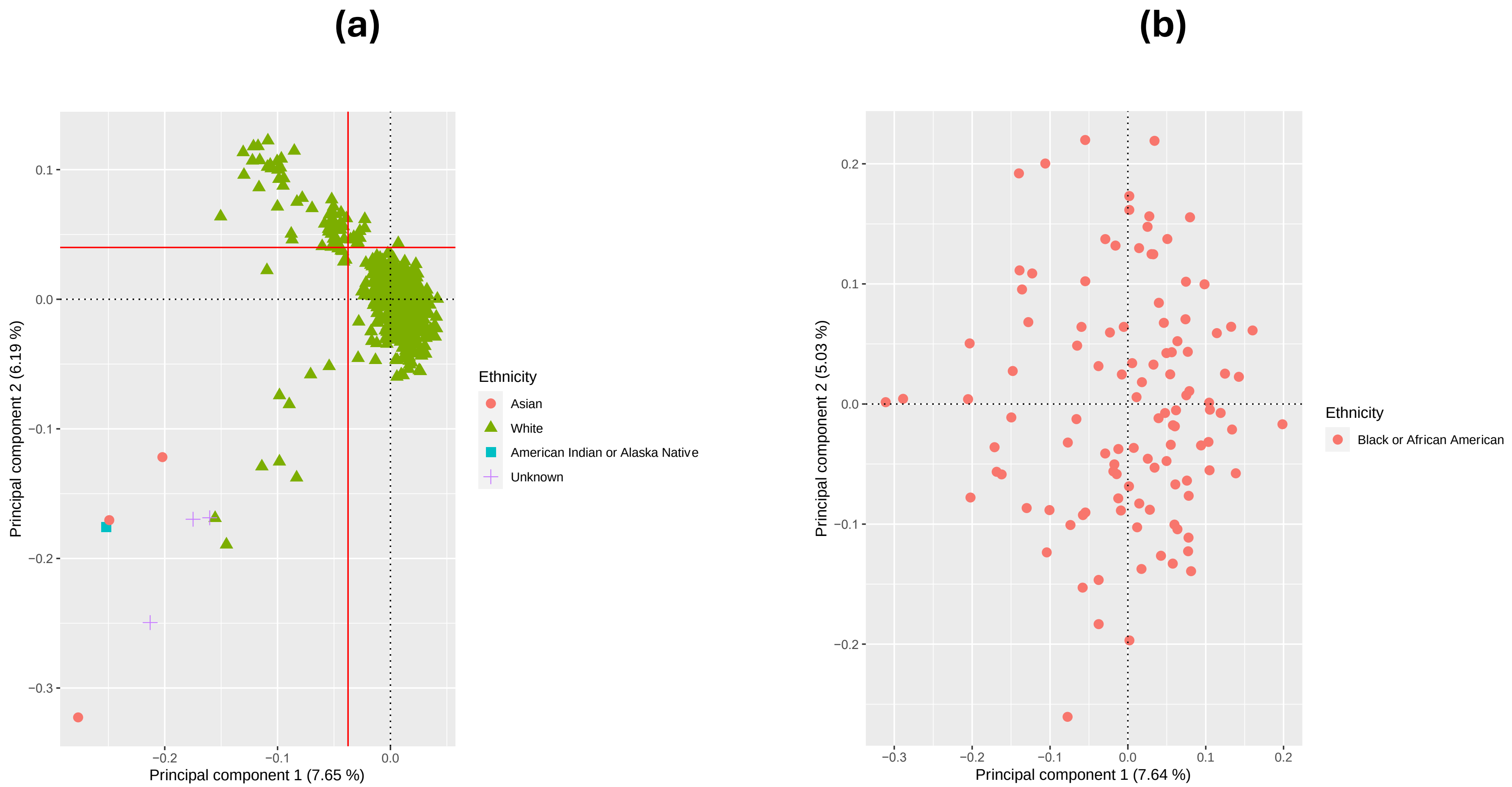

Supplementary Figure 2. Identification of European ancestry and African American individuals from the GTEx Project based on principal components analysis of the genetic relationship matrix and self-reported ethnicity. (a) Plot of the first two principal components derived from the genetic relationship matrix for the 777 individuals with heterozygosity between 0.045 and 0.050. A total of 88 individuals of outlying ancestry were excluded based on the criteria PC1 <-0.0375 or PC2 >0.04 (marked by red lines). Consequently, 689 European ancestry individuals were retained for downstream analyses. (b) Plot of the first two principal components derived from the genetic relationship matrix for the 111 individuals with heterozygosity between 0.060 and 0.080. No individuals of outlying ancestry were identified, and consequently 111 African American individuals were retained for downstream analyses.

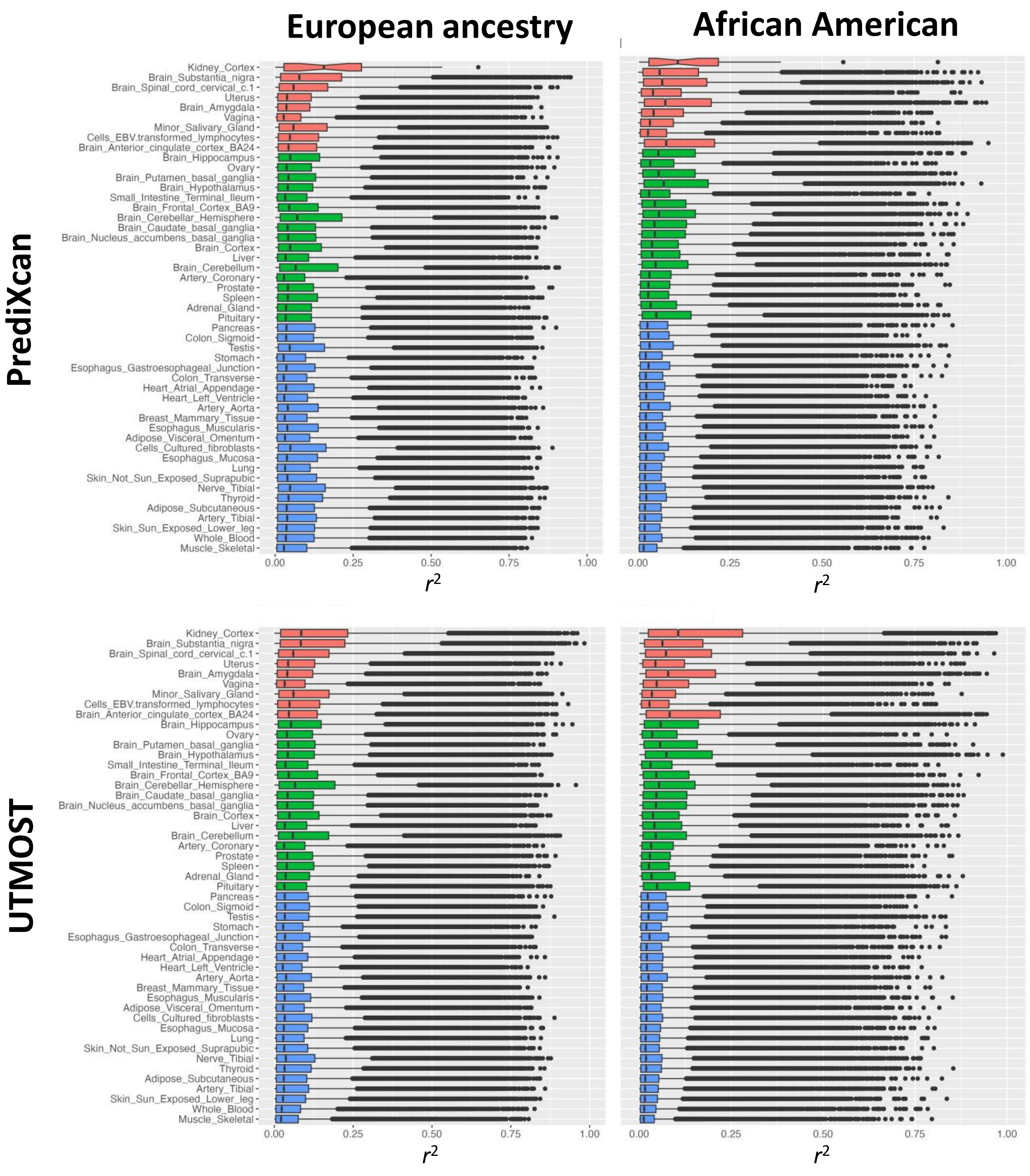

Supplementary Figure 3. Tissue-specific prediction accuracy of gene expression imputation models for the cross-tissue (UTMOST) and single tissue (PrediXcan) approaches in European ancestry and African American test datasets. Gene expression data were interrogated for 49 tissues for 38,539 autosomal genes. Prediction accuracy was assessed by the squared Pearson correlation (r^2) between imputed and observed gene expression.

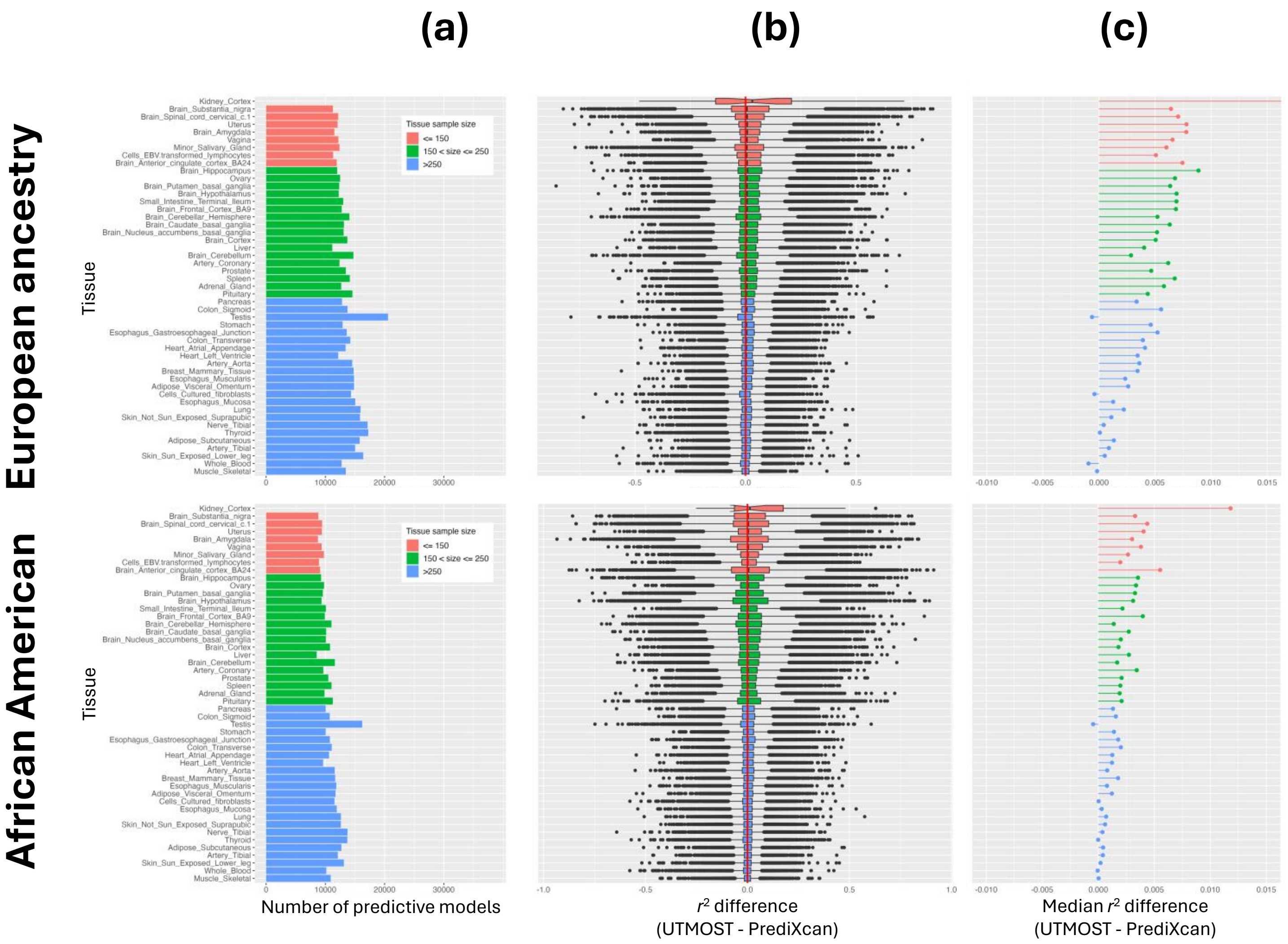

Supplementary Figure 4. Tissue-specific improvement in prediction accuracy of gene expression imputation models for the cross-tissue (UTMOST) approach over the single tissue (PrediXcan) approach in European ancestry and African American test datasets. Gene expression data were interrogated for 49 tissues for 38,539 autosomal genes. (a) Number of trained gene models that were predictive trained with both approaches. (b) Distribution of difference in squared Pearson correlation (r^2) between imputed and observed gene expression for UTMOST over PrediXcan. (c) Median difference in squared Pearson correlation (r^2) between imputed and observed gene expression for UTMOST over PrediXcan. Note that for kidney cortex in the European ancestry test dataset, the median increase in r^2 for UTMOST over PrediXcan was 0.031 (not shown for clarity of presentation).
